# Nitric Oxide Induces Autophagy in *Triticum aestivum* Roots

**DOI:** 10.3390/antiox12091655

**Published:** 2023-08-22

**Authors:** Farida Minibayeva, Anastasia Mazina, Natalia Gazizova, Svetlana Dmitrieva, Anastasia Ponomareva, Daniya Rakhmatullina

**Affiliations:** Kazan Institute of Biochemistry and Biophysics, FRC Kazan Scientific Center, Russian Academy of Sciences, 420111 Kazan, Russia; abmazina@gmail.com (A.M.); s_dmitrieva@list.ru (S.D.); na.ponomareva@mail.ru (A.P.); rahmatullina@kibb.knc.ru (D.R.)

**Keywords:** plant, nitric oxide, spermine, autophagy, energy metabolism

## Abstract

Autophagy is a highly conserved process that degrades damaged macromolecules and organelles. Unlike animals, only scant information is available regarding nitric oxide (NO)-induced autophagy in plants. Such lack of information prompted us to study the roles of the NO donors’ nitrate, nitrite, and sodium nitroprusside in this catabolic process in wheat roots. Furthermore, spermine, a polyamine that is found in all eukaryotic cells, was also tested as a physiological NO donor. Here, we show that in wheat roots, NO donors and spermine can trigger autophagy, with NO and reactive oxygen species (ROS) playing signaling roles based on the visualization of autophagosomes, analyses of the levels of NO, ROS, mitochondrial activity, and the expression of autophagic (*ATG*) genes. Treatment with nitrite and nitroprusside causes an energy deficit, a typical prerequisite of autophagy, which is indicated by a fall in mitochondrial potential, and the activity of mitochondrial complexes. On the contrary, spermine sustains energy metabolism by upregulating the activity of appropriate genes, including those that encode glyceraldehyde 3-phosphate dehydrogenase GAPDH and SNF1-related protein kinase 1 SnRK1. Taken together, our data suggest that one of the key roles for NO in plants may be to trigger autophagy via diverse mechanisms, thus facilitating the removal of oxidized and damaged cellular constituencies.

## 1. Introduction

Autophagy is a highly conserved process that involves the intracellular degradation of damaged, oxidized, or redundant macromolecules and organelles [1]. The accumulation of damaged or unwanted cellular constituents can present a threat to cellular metabolism under stress conditions and with aging. Double membrane vesicles, the so-called autophagosomes, surround unwanted components and deliver them to the vacuole (or lysosome) for subsequent degradation. Therefore, autophagy plays a significant role in the response of plants to starvation, drought, salinity, and attack by pathogens and is associated with both the survival of individual cells and the whole organism on the one hand, and on the other, programmed cell death. During plant growth and development, autophagy is necessary for seed germination and vascular cell formation. In addition, autophagy is involved in the processes of aging, organogenesis, and the biogenesis of plant vacuoles. The formation of autophagosomes is controlled by more than 40 autophagic (*ATG*) genes. The expression and the activity of the resulting gene products can be influenced by stress factors and the stage of growth and development of an organism [2]. Therefore, deciphering the mechanisms that regulate autophagy can increase our knowledge about the strategies used by plants to resist stress.

Typical effects of stress on plants include the formation and accumulation of reactive oxygen species (ROS), such as superoxide anion radical and H_2_O_2_, and reactive nitrogen species (RNS), such as nitric oxide (NO) and peroxynitrite. The pathways for generating NO in plants are more diverse than those in animals. At present, the so-called “reducing” and “oxidizing” pathways are considered to be the two main ways that plants synthesize NO, although the L-arginine-dependent source of NO (NOS-like activity) has been also reported [3]. The first pathway involves a reduction in nitrates/nitrites in the leaves and roots to NO with the participation of nitrate reductase and nitrite reductase [4]. The first step is the one-electron reduction in nitrates to nitrites by a plasma membrane nitrate reductase using nicotinamide adenine dinucleotide (NAD) (phosphate) + hydrogen (H) (NAD(P)H) as a reducing agent. In the second step, NO is formed from nitrite by a nitrite NO reductase localized in plastids. Nitrate/nitrite-dependent NO synthesis is involved in stomatal closure in response to ABA (abscisic acid), the auxin-mediated formation of lateral root primordia during abiotic stresses, and the transition to flowering [5]. The second or “oxidizing” pathway involves the metabolism of arginine through the arginase pathway and the synthesis of the regulatory polyamines, in particular spermidine and spermine [6]. Polyamines can form NO as a result of the activity of arginase and arginine decarboxylase. Polyamines are a large and widely occurring group of low molecular weight nitrogen-containing compounds. They occur in cells as organic polycations and possess high biological activity; the most common are putrescine, spermidine, and spermine [7]. In plants, polyamines play an essential role in cell proliferation and membrane stabilization, xylem formation, the suberinization and lignification of cell walls [8], the regulation of gene expression [9], and general cellular adaptation to stress [10]. Polyamines are involved in plant responses to drought, salinity, mineral deficiency, low temperatures, injury, heavy metals, ozone, paraquat, and UV-B radiation [11]. Typically, more stress-tolerant species demonstrate a greater capacity to increase the level of polyamines in response to stress factors [12]. However, NO-mediated effects of polyamines have been less studied.

Contradictory data exist on the role of NO in the regulation of autophagy. Initially, NO was thought to slow down or prevent autophagy in animal cells, but recently, more evidence is starting to emerge that NO can induce autophagy. It has been demonstrated that NO is involved in the signaling cascade Ataxia-Telangiectasia Mutated/Liver Kinase B1/AMP-activated Protein Kinase/Tuberous sclerosis complex 2 (ATM/LKB1/AMPK/TSC2). Activation of this cascade inhibits mammalian target of rapamycin complex 1 (mTORC1) and induces autophagy [13]. Interestingly, in the unicellular alga *Chlamydomonas reinhardtii*, exposure to high light causes NO emission and autophagic-induced cell death. The role of autophagy was supported by an increase in the content of the ATG8 protein, a marker protein of autophagy, and the upregulation of other *ATG* genes [14]. Autophagy was suppressed in the presence of the NO acceptor 2-4-carboxyphenyl-4,4,5,5-tetramethylimidazoline-1-oxyl-3-oxide (cPTIO). Furthermore, co-treatment of cells with H_2_O_2_ and NO donors enhanced the induction of autophagy and led to cell death after 24 h, and again the effect was eliminated by cPTIO [14]. Therefore, it seems likely that the triggering of autophagy is controlled by a synergistic interaction of ROS and RNS [15].

Unfortunately, for plants, only scant information is available on the possible induction of autophagy by NO and polyamines. It has been demonstrated that NO accumulation represses autophagy and facilitates necrotic cell death in tobacco cells exposed to a toxin isolated from the pathogenic fungus *Alternaria alternata* [16]. Repression of NO by cPTIO keeps the autophagic cascade switched on during prolonged exposure to the necrotrophic toxin. In our preliminary short communication, we visualized autophagosomes in the cells of intact wheat roots following the exogenous application of 10 μM spermine and suggested that this is mediated by intracellular NO and H_2_O_2_ production [17]. The aim of the present study was to elucidate the possible roles of NO and ROS in autophagy in the cells of wheat roots. First, the induction of autophagy was evaluated by the visualization of autophagosomes in wheat roots following treatment with the NO donors’ nitrate (KNO_3_), nitrite (KNO_2_), sodium nitroprusside (SNP), and spermine, and in tobacco leaves transfected with mRFP-TaATG8c and treated with spermine. In addition, the expression of *ATG* genes was assessed by quantitative polymerase chain reaction (qPCR) in wheat roots. Second, we determined the levels of NO and ROS in roots treated with NO donors and spermine. Third, we analyzed the energy status in the roots by analysis of mitochondrial membrane potential (ΔΨ_m_), mitochondrial ultrastructure, the activity of mitochondrial complexes, and the expression of the genes encoding the enzymes of energy metabolism GAPDH (glyceraldehyde 3-phosphate dehydrogenase) and SnRK1 (SNF1-related protein kinase 1). Here, we show that NO donors and polyamine spermine induce autophagy in wheat roots, with NO and ROS playing signaling roles. Results suggest that, depending on the source of NO, energy deficit may or may not be a prerequisite for autophagy in plants.

## 2. Materials and Methods

### 2.1. Plant Material, Growth Conditions, and Treatments

Seedlings of spring wheat (*Triticum aestivum* L.) cv. Kazanskaya Jubileinaya were grown hydroponically in distilled water for 4 d at 22 °C in a growth chamber with a 12 h-light/dark photoperiod at a light intensity of 150 μmol photons m^−2^ s^−1^. Intact roots of wheat seedlings were incubated in solutions with the NO donors 10 mM KNO_3_, 1 mM KNO_2_, 10 μM SNP, and also 1, 10, and 100 μM spermine for 3 to 12 h, depending on the experimental design. Plants of *Nicotiana benthamiana* were grown in pots with soil at 60% relative humidity for 6–7 weeks in the same temperature and light conditions as the wheat. Leaf discs of *N. benthamiana* were immersed with 10 μM spermine, vacuum infiltrated, and then incubated in Petri dishes for 3 h with gentle rotation.

### 2.2. Visualization of NO, ROS, and Autophagosomes, and the Assessment of Cell Viability and Mitochondrial Membrane Potential

Longitudinal sections of root tips taken from the extension zone (0.8 cm from the apex) were stained with fluorescent dyes. The visualization of NO was performed using 4-amino-5-methylamino-2’,7’-difluorofluorescein diacetate (10 μM DAF-FM, Invitrogen, Carlsbad, CA, USA, λ_ab_ 495 nm/λ_em_ 515 nm) [18]. The specificity of DAF-FM staining was confirmed by a significant decrease in fluorescence following the application of the NO scavenger 0.5 mM cPTIO. For the visualization of autophagosomes, cells were stained with 1 μM LysoTracker Red DND 99 (Invitrogen, Carlsbad, CA, USA, λ_ab_ 577 nm/λ_em_ 590 nm) [17]. The assessment of cell viability was performed by staining dead cells with propidium iodide (1 μM PI, Sigma, λ_ab_ 485 nm/λ_em_ 610 nm) [19]. The mitochondrial membrane potential was assessed using the voltage-dependent fluorescent dye tetramethylrhodamine methyl ester (1 μM TMRM, Sigma, Shanghai, China, λ_ab_ 543 nm/λ_e_m 573 nm), which accumulates in the matrix of energized mitochondria [20]. Fluorescence images were visualized using a confocal microscope LSM-510 META (Carl Zeis, Jena, Germany). Digital quantifications of the relative fluorescence of LysoTracker Red and TMRM using ImageJ version 1.53 are presented in the Supplementary Materials.

### 2.3. Determination of NO, H_2_O_2_, and Lipid Peroxidation

Determination of NO in the roots was carried out by electron paramagnetic resonance (EPR) using the NO trap sodium diethyldithiocarbamate (DETC) [21]. The EPR signal of mononitrosyl iron complexes with DETC in roots treated with spermine was recorded on an EMX X-band spectrometer (Bruker, Bremen, Germany) at a temperature of 77 °K.

The H_2_O_2_ content in the supernatants of root homogenates was determined spectrophotometrically using the xylenol orange method (Sigma, Shanghai, China) using a Lambda-25 spectrophotometer (PerkinElmer, Waltham, CT, USA, λ_560_). The concentration of H_2_O_2_ was calculated using a calibration curve [22]. Lipid peroxidation in the soluble fraction of the homogenate was assessed spectrophotometrically by measuring the content of TBA-reactive products (λ_532_) [23].

### 2.4. Membrane Permeability and Oxygen Consumption by Roots

Membrane permeability was evaluated by the leakage of electrolytes from roots using a conductivity meter, Cond 7310 (WTW, Weilheim, Germany), and the index of membrane stability (IMS) was calculated as a percentage of the total yield of electrolytes [20].

The rates of oxygen consumption by roots were measured by the Warburg manometric method. Roots (150 mg) were immersed in the corresponding solutions (3 mL) in Warburg vials. Respiration rates were measured during 3 h [20].

### 2.5. Extraction and Immunodetection of Autophagic Proteins

Proteins were isolated by phenol extraction according to the protocol [24] from the root tips after fixing them in liquid nitrogen. The final pellets were dried under air in a hood for 1 h and re-dissolved in 4% (*w*/*v*) SDS, and protein contents were determined using a Pierce BCA Protein Assay Kit (Thermo Scientific, Waltham, MA, USA).

Protein electrophoresis was performed in a 4% polyacrylamide concentrating gel at 40 V and a 10% separating gel at 120 V in a Mini-PROTEAN Tetra Cell chamber (Bio-Rad, Hercules, CA, USA) [25]. In all samples, 10 µg of protein was applied to the track. Proteins separated electrophoretically were transferred onto polyvinylidene difluoride (PVDF) membranes by semi-dry blotting using an SDS-PAGE transfer buffer at 150 mA for 1 h. Membranes were incubated with rabbit polyclonal to APG8A/ATG8A (1:2000, ab77003, Abcam, Boston, MA, USA) or rabbit polyclonal to ATG4B (1:500, ab69937, Abcam, Boston, MA, USA). α-Tubulin was used as a loading control. To visualize autophagic proteins, blots were incubated with secondary anti-rabbit IgG antibodies conjugated with horseradish peroxidase (1:5000, ab205719, Abcam, Boston, MA, USA), a chemiluminescent substrate (0.1 M Tris-HCl, luminol, p-coumaric acid), and 30% hydrogen peroxide. Blots were then scanned using an imaging system (ChemiDoc MP, BioRad, Hercules, CA, USA). Digital quantifications of the western blots using ImageLab are presented in the Supplementary Materials.

### 2.6. Isolation of Mitochondrial Proteins, BN-PAGE, and In-Gel Activity Staining of Mitochondrial Complexes

Mitochondria were isolated from the roots by differential centrifugation followed by Percol gradient centrifugation [26]. Mitochondrial proteins were solubilized in a buffer (30 mM HEPES pH 7.4, 150 mM potassium acetate, 10% glycerol, 2 mM PMSF, and digitonin), incubated for 20 min on ice, and then centrifuged at 18,000× *g* for 30 min [27]. Coomassie solution (5% Coomassie Brilliant Blue G-250 in 750 mM aminocaproic acid) was added to the supernatants, and the samples were immediately loaded onto blue-native gradient (4–13%) gels. BN-PAGE was performed according to [28].

The detection of mitochondrial oxidative phosphorylation (OXPHOS) complexes was carried out after Blue Native Polyacrylamide Gel Electrophoresis (BN-PAGE) by staining gels with Coomassie Brilliant Blue R-250. The NativeMARK^TM^ Unstained Protein Standard markers (Invitrogen, Carlsbad, CA, USA) and the Scion Image program were used to determine the molecular masses of the complexes after gel scanning using an imaging system (ChemiDoc MP, BioRad, Hercules, CA, USA). The in-gel activity staining of the complexes was assessed using dyes and substrates specific to each complex [29]. Sodium nitroprusside was added to the gel strips before activity staining for 1 h. Digital quantifications of the BN-PAGE bands using ImageJ version 1.53 are presented in the Supplementary Materials.

### 2.7. Ultrastructure of Root Cells

For transmission electron microscopy, the intact roots from 10–15 randomly chosen wheat seedlings were incubated by gentle shaking in working solutions for 3 h. Root slices 1–2 mm thick from the root elongation zone were initially fixed with a 2.5% glutaraldehyde solution (1.5 h) and then post-fixed with 1% osmium tetroxide in 0.1 M Na phosphate buffer, pH 7.4, for 2 h, and then the samples were processed as described previously [20]. The preparations were examined using a transmission electron microscope, Jem-1200 EX (Jeol Ltd., Akishima, Japan).

### 2.8. Transient Expression of mRFP-ATG8 Protein

The full-lengths cDNA of mRFP and TaATG8c were generated by PCR using specific primers (Table S1). ATG8 amplicons digested with appropriate restriction enzymes were inserted downstream and in-frame with the mRFP ORF into the pLH7000Δ binary vector under dual 35STMV promoters. The recombinant plasmid was checked by sequencing, and the digestion of restriction enzymes and transformed into *Agrobacterium tumefaciens*. Leaves of *N. benthamiana* plants were agroinfiltrated and, after 3–4 d localization of mRFP-ATG8c (λ_ab_ 488 nm/λ_em_ 525 nm), were visualized using a fluorescence microscope, LSM 510 META (Zeiss, Göttingen, Germany).

### 2.9. Analysis of Gene Expression by Quantitative Real-Time PCR

Intact roots of wheat seedlings were incubated in solutions with the NO donors 10 mM KNO_3_, 1 mM KNO_2_, 10 μM SNP, and also spermine with concentrations of 1, 10, and 100 μM for 3, 6, and 12 h. At each time point, total RNA was isolated from the roots using ExtractRNA (Evrogen, Moscow, Russia) according to the manufacturer’s protocols. RNA concentration and purity were assessed using a NanoDrop^®^ ND-1000 spectrophotometer (Thermo Scientific, Waltham, MA, USA), and the integrity was verified by 1% agarose gel electrophoresis. A reverse transcription (RT) reaction was performed using a C1000 Touch™ Thermal Cycler (Bio-Rad, Hercules, CA, USA) with an MMLV RT kit (Evrogen, Moscow, Russia) in a reaction volume of 25 μL, according to the standard manufacturer’s protocol.

Real-time qPCR was performed using a CFX Connect Real-Time PCR Detection System (Bio-Rad, Hercules, CA, USA). The templates were amplified three times at 95 °C for 3 min, followed by 40 cycles of amplification (94 °C for 10 s and 55/65 °C for 40 s). ADP ribosylation factor (*TaARF*) and RNase L inhibitor-like protein (*TaRLI*) genes were used as reference genes [30]. Sequences of primers for *TaGAPDH* were used from [31], *TaATG 5*,*7*,*10* from [32], and *TaATG 3a*, *5a*, *12b*, *13a* from [33]. The primers used for *TaSnRK1* and other *TaATG* genes are shown in Supplementary Table S1. Differences in gene expression were normalized (∆∆Cq) using Bio-Rad CFX Maestro version 2.3 (Bio-Rad, Hercules, CA, USA). The relative level of transcripts in the control was considered as one unit.

### 2.10. Statistics

All experiments were performed with at least three biological and six analytical replicates. The data were expressed as the mean ± SD. For Table 1 and Figures S3–S6, statistical analysis was performed using the two-tailed Student’s *t*-test in Microsoft 365 (Excel). For gene expression, statistical differences were analyzed by ANOVA following the test for normality with the Shapiro–Wilk test using the software package CFX Maestro version 2.3 (Bio-Rad, Hercules, CA, USA). Statistically significant differences from the control are marked with an asterisk, where (*) *p* ≤ 0.05. For spermine treatments, statistical differences in gene expression were evaluated by the two-way analysis of variance ANOVA (Originpro version 9, Northampton, MA, USA), with time and concentration as factors. Differences were significant at (*) *p* ≤ 0.05 and (**) *p* ≤ 0.01 (Table S2).

## 3. Results

### 3.1. NO Donors, Spermine-Induced Autophagosome Formation, and ATG Gene Expression in Wheat Roots

**Figure 1:**
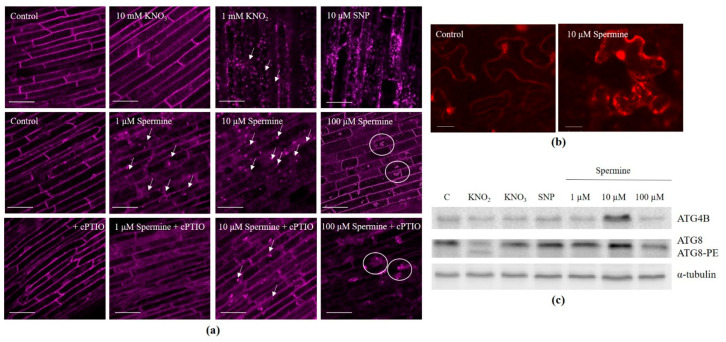
Formation of autophagosomes and the expression of autophagic proteins in the wheat roots treated with NO donors and spermine: (**a**) representative confocal microscopy images of autophagosomes obtained using the fluorescent probe LysoTracker Red (λ_ab_ 577 nm/λ_em_ 590 nm, bar = 50 μm). The arrows indicate autophagosomes, the circles indicate LysoTracker Red-positive conglomerates. Digital quantification of the relative LysoTracker Red fluorescence is presented in Figure S3. (**b**) mRFP-TaATG8c-dependent fluorescence corresponding to the presence of autophagosomes in *Nicotiana benthamiana* leaves after exposure to 10 μM of spermine; bar = 20 μm. (**c**) representative western blots of the TaATG4B and TaATG8 proteins from wheat roots after 3 h of treatment with NO donors and spermine. α-Tubulin was used as a loading control. Digital quantification of the western blot changes is presented in Figure S4. Data represent typical images of at least three replicates.

Autophagosomes were visualized as bright dots by staining with LysoTracker Red in the roots treated with KNO_3_, KNO_2_, and spermine at concentrations of 1 and 10 µM for 3 h (Figure 1a). The NO trap cPTIO decreased the number of spermine-induced autophagosomes (Figure S1). Using transmission electron microscopy, autophagosomes were visualized in the cytoplasm and central vacuole following treatment of the roots with spermine at concentrations of 1 and 10 µM (Figure S2). The application of SNP and 100 μM spermine to the roots led to the appearance of large LysoTracker-positive conglomerates (Figure 1a). After long exposure to SNP (16 h) and spermine (12 h) at high concentrations, conglomerates were preserved, while in roots treated with KNO_3_, KNO_2_, and spermine at more physiological concentrations, the numbers of autophagosomes were significantly reduced (Figure S3).

The activity of numerous ATG proteins is required to form autophagosomes, and ATG8 protein is considered a marker for the detection of autophagosomes [2]. Thus, to confirm the formation of autophagosomes in response to the elevated levels of NO, we used tobacco leaves transfected with fluorescently labeled wheat ATG8 (mRFP-TaATG8c). For this purpose, *Agrobacterium tumefaciens* cells were transformed with plasmid constructs containing the open reading frame of the wheat ATG8c gene with an inserted sequence of the mRFP fluorescent protein. To check that the chimeric protein mRFP-ATG8c was produced in *Nicotiana benthamiana* cells, the level of fluorescence inherent in the reporter protein and intracellular localization were analyzed in infiltrated leaves. After incubation of the cut leaf disks of the transformed plants in solutions with 10 μM spermine, multiple dots corresponding to autophagosomes containing the mRFP-ATG8 protein were visualized. This effect was most clearly observed following 3 h of treatment with 10 μM spermine (Figure 1b), confirming the formation of autophagosomes.

To immunodetect the accumulation of the proteins ATG4 and ATG8 in the roots exposed to NO donors and spermine, antibodies against ATG4B and ATG8 were used. The highest level of ATG4B was observed after 10 μM spermine treatment for 3 h (Figure 1c and Figure S4a), while high levels of ATG8 were also present after most of the treatments, except 100 μM spermine (Figure 1c and Figure S4b). Interestingly, following 3 h KNO_2_ treatment, ATG8 was visualized in delipidated and lipidated (ATG8-PE) forms.

**Figure 2:**
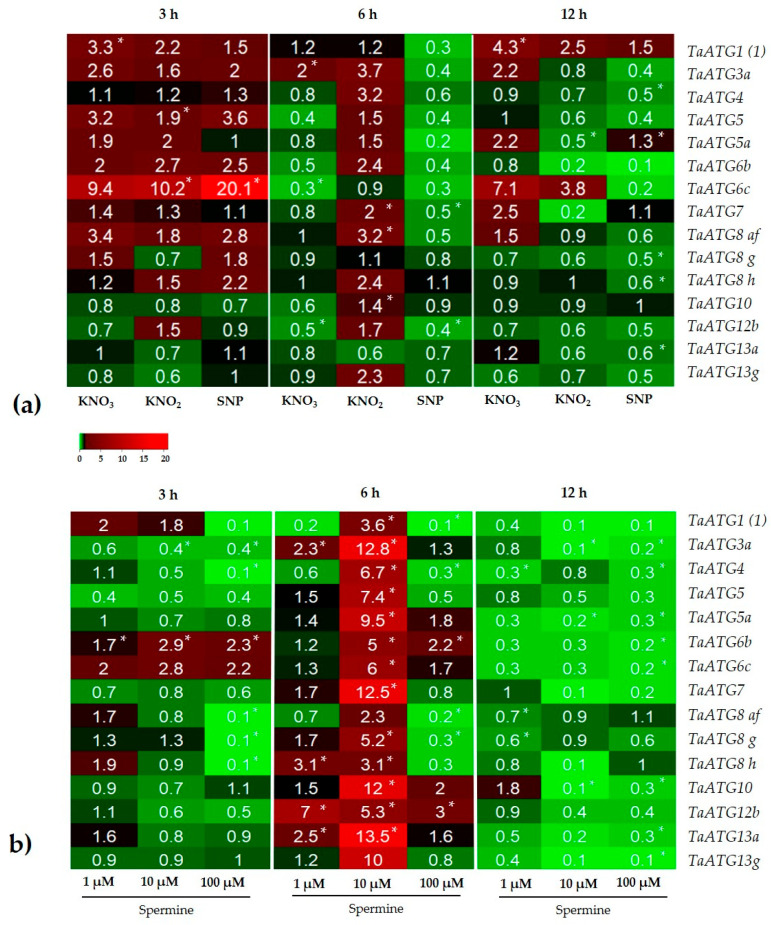
qPCR analysis of the time-course of *ATG* gene expression in wheat roots treated with (**a**) NO donors; (**b**) spermine. Downregulated gene expression is indicated by green, while upregulated gene expression is indicated by red. An asterisk (*) denotes a significant difference between the control and treatments according to ANOVA (*p* < 0.05, *n* = 6). For (**b**) statistically significant differences in the effects on gene expression, the the time of exposure and the concentrations of spermine were derived from a two-way ANOVA (Table S2). Differences were significant at (*) *p* ≤ 0.05, (**) *p* ≤ 0.01.

Real-time PCR analyses of the activity of several key *ATG* genes that control various stages of autophagosome formation indicated that genes such as *TaATG1*, *TaATG6*, and *TaATG8 af* display high transcription levels in roots treated with NO donors after 3, 6, and 12 h (Figure 2a). Comparing the effects of the NO donors demonstrated different time-dependences for the upregulation of *ATG* genes, e.g., KNO_3_ caused the upregulation of *ATG* genes mainly after 3 and 12 h, while KNO_2_ after 3 and 6 h. SNP only upregulated *ATG* genes after 3 h and caused the downregulation of all *ATG* genes after 6 and 12 h (Figure 2a). In response to spermine, treatment expression of *ATG* genes followed a bell-shaped response. During short (3 h) and long (12 h) treatments of the roots with spermine, most *ATG* genes were downregulated, except isoforms of *TaATG6* after 3 h (Figure 2b). Interestingly, treatment of the roots with spermine for 6 h significantly upregulated *ATG* genes (Table S2). The genes showing the greatest increase were *TaATG3a*, *TaATG5*, *TaATG7*, *TaATG10*, *TaATG13a*, and *TaATG13g* (Figure 2b).

### 3.2. Accumulation of NO and ROS

**Figure 3:**
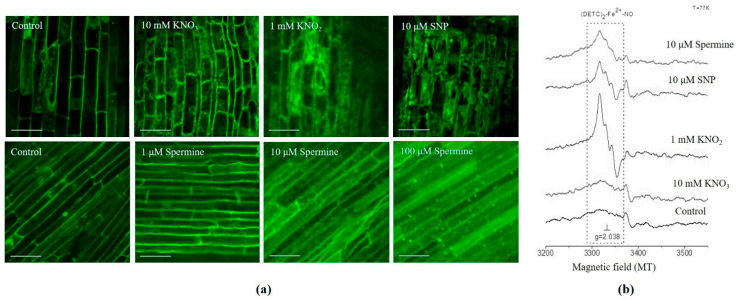
NO production in wheat roots treated with NO donors and spermine: (**a**) representative confocal microscopy images of NO-dependent fluorescence obtained using the fluorescent probe DAF-FM (λ_ab_ 495 nm/λ_em_ 515 nm, bar = 50 μm); (**b**) EPR spectra of (DETC)_2_-Fe^2+^-NO signal (dotted line). Data represent typical images of at least three replicates.

Exposing roots to NO donors and spermine significantly changed their redox status. The accumulation of NO was detected by an increase in the fluorescence level of DAF-FM, a NO-specific dye (Figure 3a). The highest NO production was observed with KNO_2_, SNP, and spermine at concentrations of 10 and 100 μM. Furthermore, increased NO formation in the roots after treatment for 3 h with KNO_2_, SNP, and 10 μM spermine, which was confirmed using EPR. The formation of the characteristic paramagnetic spectra of the triplet structure of Fe^2+^ complexed with NO was detected using the NO-specific trap DETC (Figure 3b). The most pronounced spectrum was induced by KNO_2_, SNP, and 10 μM spermine. Markers of oxidative stress were also elevated. The H_2_O_2_ content was doubled in roots treated for 6 h with KNO_2_, SNP, and spermine at 1 and 10 μM, and increased three times after 100 μM spermine treatment (Table 1).

KNO_2_, SNP, and 100 μM spermine elevated the level of lipid peroxidation, which is estimated as MDA content, indicating the development of oxidative stress (Table 1). Indirectly, this was confirmed by changes observed in plasma membrane permeability. Almost all treatments reduced the index of membrane stability (IMS), except for 1 μM spermine (Table 1). Interestingly, the increased plasma membrane permeability induced by KNO_3_ and 10 μM spermine (Table 1) was not accompanied by a decrease in cell viability (Figure S3).

### 3.3. Mitochondrial Activity and the Expression of Genes of Energy Metabolism

**Figure 4:**
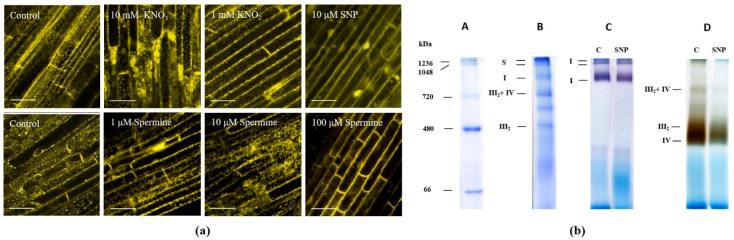
Mitochondrial membrane potential and in-gel activity of mitochondrial OXPHOS complexes and supercomplexes in NO donors and spermine-treated wheat roots: (**a**) representative confocal microscopy images of mitochondrial membrane potential-dependent fluorescence obtained using the fluorescent probe TMRM (λ_ab_ 543 nm/λ_em_ 573 nm, bar = 50 μm). Digital quantification of the relative TMRM fluorescence is presented in Figure S5. (**b**) Representative BN-PAGE images of mitochondrial OXPHOS complexes and supercomplexes. Lane A—native protein markers, Coomassie staining. Lane B—OXPHOS complexes and supercomplexes: I—complex I; III_2_—dimer of complex III; S—supercomplexes I + III_2_ + IV; III_2_ + IV, Coomassie staining. Lane C—in-gel activity of complex I, staining by nitro blue tetrazolium (NBT). Lane D—in-gel activity of complex IV, complex III_2,_ and supercomplex III_2_ + IV; staining by diaminobenzidine (DAB). C—control, SNP—sodium nitroprusside. Digital quantifications of the relative activity of complexes IV and III_2_ and supercomplex III_2_ + IV are presented in Figure S6. Data represent typical images of at least three replicates.

The effects of NO donors and spermine on mitochondrial activity were examined; in particular, mitochondrial potential, respiration rates, and the activity of mitochondrial complexes. High ΔΨ_m_ was visualized as bright TMRM fluorescence dots in response to KNO_3_ and spermine at concentrations of 1 and 10 μM (3 h). KNO_2_, SNP, and 100 μM spermine strongly decreased ΔΨ_m_ (Figure 4a and Figure S5) and reduced respiration rates after 3 h (Table 1). Using BN-PAGE, we detected mitochondrial electron transport chain (ETC) protein complexes and supercomplexes by staining with Coomassie Brilliant Blue R-250 (Figure 4b). Analysis of the in-gel activity of complexes III and IV, after their separation by BN-PAGE and staining with specific substrates and dyes, revealed that the activity of complexes III2 and IV, as well as supercomplex III2 + IV, markedly decreased after exposure of the gels to SNP for 1 h (Figure 4b and Figure S6).

**Figure 5:**
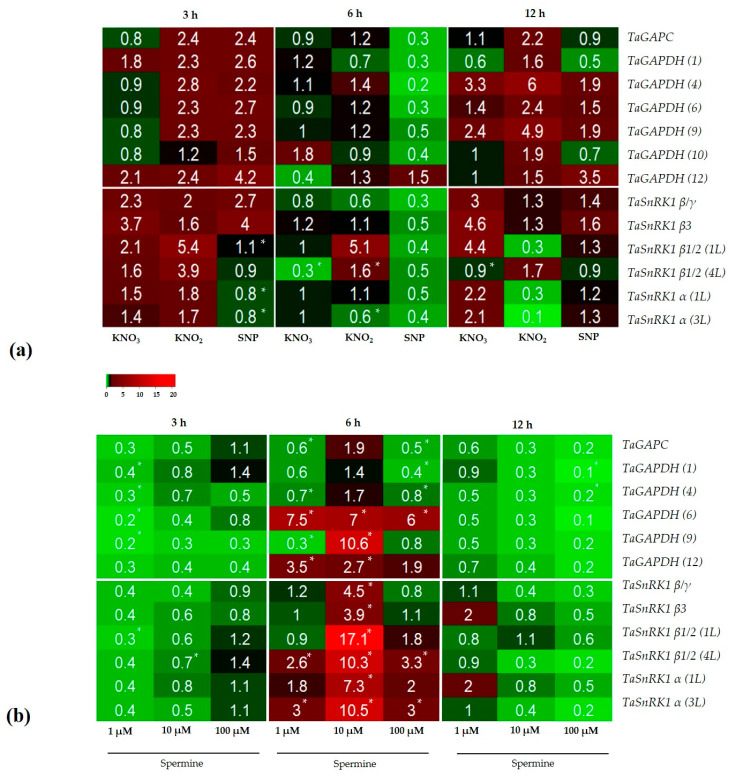
qPCR analysis of the time-course of energy-related gene expression in wheat roots treated with (**a**) NO donors; (**b**) spermine. Downregulated gene expression is indicated by green, while upregulated gene expression is indicated by red. An asterisk (*) denotes a significant difference between the control and treatments according to ANOVA (*p* < 0.05, *n* = 6). For (**b**) statistically significant differences in the effects on gene expression, the time of exposure and the concentrations of spermine were derived from a two-way ANOVA (Table S2). Differences were significant at (*) *p* ≤ 0.05, (**) *p* ≤ 0.01.

NO donors and spermine affected the expression of genes, encoding isoforms of the glycolytic enzyme GAPDH and subunits of AMP-dependent protein kinase SnRK1 in a time-dependent way. For NO donors, an inverted bell curve was observed with higher gene expression after 3 and 12 h and lower gene expression after 6 h, especially for KNO_3_ and KNO_2_ (Figure 5a). The greatest increases in expression were observed for TaSnRK1 β/1 (1 L) (5–6 times). For spermine, the time-dependences of the transcription of the analyzed genes were similar to ATG genes, displaying a bell-shaped pattern. During short- (3 h) and longer- (12 h) term treatment of the roots with spermine, most GAPDH and SnRK1 genes were downregulated, except TaSnRK1 β/3 and TaSnRK1 α (1 L and 3 L), after 12 h (Figure 5b). In contrast, the levels of the GAPDH and SnRK1 transcripts greatly increased after 6 h exposure of the roots to spermine. For example, in response to 10 µM spermine, the levels of transcripts of TaGAPDH (6), TaGAPDH (9), and TaGAPDH (12) were statistically increased. All SnRK1 genes were highly upregulated in response to 10 µM spermine, e.g., the expression level of TaSnRK1 β/1 (1 L), TaSnRK1 β1/2 (4 L) isoform, and TaSnRK1 α (3 L) increased by more than 10-fold.

## 4. Discussion

### 4.1. NO Donors, Spermine, and Autophagy

Although it is widely recognized that autophagy plays significant roles in the response of plants to stresses and is associated with survival and/or programmed cell death, little is known about NO-dependent mechanisms that regulate this catabolic process. Here, we show that in wheat roots, NO donors and spermine can trigger autophagy, with NO and ROS playing signaling roles.

Among NO donors, nitrite is the most effective inducer of autophagy in wheat roots. The generation of NO is confirmed by fluorescence dye staining (Figure 3a) and EPR spectra (Figure 3b) and is accompanied by the accumulation of H_2_O_2_ and an increase in lipid peroxidation (Table 1), which are indicators of oxidative stress. Furthermore, NO generation induces the formation of autophagosomes (Figure 1a and Figure S1) and the upregulation of *ATG* genes (Figure 2). Nitrate, another member of the reducing pathway of NO generation in plants, is less effective in the induction of autophagosome formation (Figure 1), although prolonged (12 h) exposure of the roots to KNO_3_ results in the appearance of autophagosomes (Figure S3). The classic NO producer SNP generates NO (Figure 3) and causes oxidative stress (Table 1) and the massive accumulation of LysoTracker Red-positive conglomerates (Figure 1a), although only a few *ATG* genes are upregulated (Figure 2a). After a long-term (16 h) treatment with KNO_2_ and SNP, cell viability decreases (Figure S2), most likely as a consequence of nitrosative and oxidative stresses. It seems that the synergistic effects of NO and ROS can effectively induce autophagy in the cells of wheat roots.

The search for a natural inducer of autophagy prompted us to test the effects of polyamines, members of the oxidizing pathway of NO generation in plants. We found that in wheat roots, spermine induces autophagosome formation, and this is significantly inhibited by the NO trap cPTIO (Figure 1a and Figure S1) and strongly suggests the involvement of NO in this process. The ability of spermine to increase NO level is confirmed by the increase in DAF-FM fluorescence (Figure 3a) and the detection by EPR of a typical NO signal with a characteristic triplet structure (Figure 3b). Further confirmation that exogenous spermine can induce autophagy in wheat roots comes from the visualization of autophagosomes using transmission electron microscopy (Figure S2), the accumulation of chimeric protein mRFP-TaATG8c in tobacco leaves exposed to spermine (Figure 1b), and the immunodetection of ATG8, a marker of autophagosome formation, by an ATG8-specific antibody (Figure 1c). Interestingly, while high levels of immunodetected ATG8 are present in most samples, only treatment of the roots with 10 μM spermine causes accumulation of both ATG4 and ATG8 (Figure 1c and Figure S4). ATG4, a cysteine-containing protease, is the only redox-sensitive ATG protein, and the interaction of this protein with ATG8 occurs during two stages of autophagosome formation, facilitating first, together with other ATG proteins, the formation of lipidated (ATG8-PE), and later the delipidation of ATG8 [2]. This post-translational modification of ATG8 is necessary for its binding and subsequent release from membranes during autophagosome formation. Although we were unable to immunodetect these two forms of ATG8 in the sample after spermine treatment, they were clearly visualized in the sample after KNO_2_ treatment (Figure 1c). Taken together, our data suggest that it is NO that mediates autophagic processes induced by spermine in the root cells of wheat seedlings.

It is the ability of polyamines to generate ROS that defines their role in plant metabolism [34,35]. The metabolism of polyamines in the cells is often mediated by activation of polyamine oxidases and results in the formation of H_2_O_2_ [36], explaining the increases in the H_2_O_2_ content (Table 1) that occur following treatment with all concentrations of spermine. ROS are known as the most important signaling molecules involved in the initiation of autophagy in plant cells [37]. For example, Zhang and co-authors demonstrated that apoplastic H_2_O_2_ mediates spermidine-induced autophagy that alleviates salt stress in cucumbers [38]. Spermidine-induced salt tolerance and autophagy were compromised when plants were pretreated with dimethylthiourea, an ROS scavenger, or diphenyleneiodonium chloride, an inhibitor of NADPH oxidase. Unfortunately, the authors did not test if the effects of spermidine are mediated by NO. However, given that polyamines induce the production of both NO and ROS, it seems likely that the role of polyamines as autophagy inducers can be mediated by the synergistic effects of RNS and ROS.

Wheat roots treated with NO donors and spermine show time-dependent changes in the activity of several key *ATG* genes that control various stages of autophagosome formation. These genes include the ATG1 protein kinase complex (*ATG1*, *ATG13a*), the ATG8—a conjugation system (*ATG8 af*, *g*, *h*; *ATG4*, *ATG7*, *ATG3a*), and the ATG12—a conjugation system (*ATG12b*, *ATG7*, *ATG10*, *ATG5*, *ATG5a*). While NO donors mainly upregulate *ATG* genes during the first 3 and 6 h of treatment (Figure 2a), spermine-mediated *ATG* gene expression displays a bell-shaped pattern. An exogenous application of spermine downregulated the expression of almost all *ATG* genes after 3 h (Figure 2b). An exception was an upregulation of genes encoding the ATG1 protein kinase complex, which is involved in the induction of phagophore formation. It seems likely that the induction of autophagy evidenced by the accumulation of autophagosomes after 3 h of exposure of the roots to spermine is mediated by the existing pool of transcripts of *ATG* genes and proteins (Figure 1b,c). Further treatment of the roots with spermine for 6 h causes a large statistically significant upregulation of most *ATG* genes (Table S2), which are involved in various stages of autophagosome formation. Specifically, the highest expression was observed for *ATG* genes encoding the proteins involved in the ATG1 protein kinase complex (*ATG1*, *ATG13a*), e.g., the level of *TaATG13a* transcripts increases more than 13 folds, and ATG8—a conjugation system, e.g., the level of *TaATG3a* and *TaATG7* transcripts, increases more than 12 folds. It is known that ATG7, ATG10, and ATG12b proteins belong to the ATG12 conjugation system, and this system is responsible for the elongation of the phagophore, which is one of the key stages of autophagosome formation [2]. Corresponding genes are also highly upregulated (Figure 2b). Therefore, it can be suggested that NO donors and spermine treatment cause the upregulation of genes, encoding the proteins involved in all stages of autophagosome formation.

### 4.2. NO Donors, Polyamine-Induced Autophagy, and Energy Status

Mitochondria, as the main energy-producing and also ROS-generating cellular organelles, play an important role in autophagy [37]. In this study, we show that the treatment of wheat roots with KNO_3_ and spermine at concentrations of 1 and 10 μM elevates the mitochondrial potential, while KNO_2_, SNP, and 100 μM spermine strongly decrease ΔΨ_m_ (Figure 4a and Figure S5) and reduce respiration rates (Table 1). We suggest that the production of NO by KNO_2_, SNP, and 100 μM spermine affects the activity of mitochondrial complexes. Indeed, analysis of the in-gel activity of mitochondrial electron transport chain (ETC) protein complexes and supercomplexes, after their separation by BN-PAGE and staining with specific substrates and dyes, demonstrate that the activity of complexes III_2_ and IV, as well as supercomplex III_2_ + IV, markedly decrease after exposure of the gels to SNP (Figure 4b and Figure S6). NO is known to affect the activity of the terminal ETC complex IV (cytochrome c oxidase) [39].

Polyamines, depending on their concentration, can change mitochondrial functioning either stimulating or suppressing activity. For example, the oxidation products of polyamines induce the uncoupling of oxidation and phosphorylation and cause the release of cytochrome *c* from mitochondria [40]. However, the respiratory competence of cardiac mitochondria through complex I in the mitochondrial ETC is increased in mice supplemented with spermidine [41]. In wheat seedlings treated with spermine at physiological concentrations (1 and 10 μM) for 3 h, mitochondria display normal round or oval shapes. They maintain typical ultrastructures with several cristae, possess dense matrices (Figure S2), and sustain high ΔΨ_m_, which is visualized as bright TMRM fluorescence (Figure 4a). Such mitochondrial activity may be associated with the effect of spermine on the oxidation–phosphorylation complexes of the mitochondrial ETC. In contrast, treating wheat roots with a high concentration of spermine damages the structural integrity of mitochondria (Figure S2), reduces the mitochondrial membrane potential (Figure 4a), and significantly reduces the rates of respiration (Table 1). These observations indicate that high concentrations of spermine can damage the structure and impair the functioning of mitochondria. Furthermore, an increase in lipid peroxidation (Table 1), the formation of cPTIO-insensitive LysoTracker-positive conglomerates (Figure 1a), and a decrease in the index of membrane stability (Table 1) suggest that at high, non-physiological concentrations polyamines cause toxicity.

It seems likely that the changes in the energy and redox states of cells induced by NO donors and spermine are accompanied by changes in the expression of genes, encoding key enzymes of energy metabolism, specifically the glycolytic enzyme GAPDH. GAPDH has a redox-sensitive cysteine in the catalytic center and can, therefore, reversibly change its own activity [42]. Moreover, this cysteine is a potential site for the S-nitrosylation of GAPDH and can, therefore, provide a link between energy metabolism, redox status, and NO. In addition to playing a role in glycolysis, GAPDH participates in several non-glycolytic functions, including autophagy [43]. In our experiments, the expressions of only a few isoforms of *TaGAPDH* genes are upregulated by KNO_2_ treatment after 3 and 6 h (Figure 5a). Following treatment of the roots with spermine, *TaGAPDH* genes were downregulated after short- and long-term exposure but were highly upregulated after 6 h (Figure 5a). GAPDH is a housekeeping protein, and, therefore, strong changes in the expression of the encoding gene probably indicate that the changes are occurring in the pool size of this protein; however, this suggestion needs further experimental confirmation.

Another energy-related protein, SnRK1, an ortholog of AMP-activated protein kinase (AMPK) in mammals, serves as a positive regulator of autophagy, in contrast to the protein kinase target of rapamycin (TOR), which is a negative regulator of autophagy [44]. The kinases SnRK1 and TOR closely interact with each other to form the regulatory core of various metabolic pathways. For example, a lack of substrates, such as sugars, causes the inhibition of TOR, the activation of SnRK1, and the induction of autophagy [45,46]. Moreover, SnRK1 can trigger autophagy via a TOR-independent pathway by phosphorylating ATG1 and facilitating its conjugation with ATG13, or directly activating ATG6 [47]. Interestingly, NO treatment only moderately increases the activity of *TaSnRK1* genes, in particular after 3 h (Figure 5a), the time when an increased expression of *TaATG6* occurs (Figure 2a).

SnRK1 is a heterotrimeric complex where catalytic α-subunits can be phosphorylated and thus affect the activity of the kinase. β subunits (SnRK1β) control kinase activity, localization, and substrate specificity. γ subunits function as a cellular energy-sensitive module of the complex. In green plants, a hybrid βγ subunit (SnRK1βγ) acts as the canonical γ subunit. It has been shown that the overexpression of the SnRK1α subunit is sufficient to provide high and specific SnRK1 activity in wild type leaf cells and transgenic plants [48]. Interestingly, the energy-sensitive βγ subunit is only moderately expressed in wheat roots after exposure to NO donors (Figure 5a) and is not expressed after 3 and 12 h of exposure to all concentrations of spermine (Figure 5b). However, after 6 h of exposure of the roots to spermine, there is a significant upregulation of the genes, encoding all *SnRK1* subunits (Figure 5b), suggesting that SnRK1 acts as a full-fledged element of the autophagic cascade.

We suggest that in the roots of wheat, the polyamine spermine at physiological concentrations (1 and 10 μM) induces autophagy via an energy-independent pathway, as evidenced by increases in the potential of mitochondrial membranes and the rates of respiration (Figure 4a, Table 1). Similar to *ATG* genes, treatment with spermine induces a bell-shaped response with time in the expression of the genes, encoding GAPDH isoforms and SnRK1 subunits, especially for spermine 10 μM (Figure 5b). Interestingly, only 1 μM of spermine retains its stimulating effect after 12 h of exposure, although this is not statistically significant (Figure 5b), despite causing the low upregulation of the genes after 6 h of exposure of the roots compared to 10 μM spermine. Thus, the effects of NO donors and spermine on the expression of energy-related genes tested here display time- and NO dose-dependency (Table S2).

## 5. Conclusions

Here, we show that in wheat roots, the exogenous application of NO donors and spermine can trigger autophagy. However, the mechanisms behind the induction of autophagy by NO remain unclear. Considering the known synergy between NO and ROS, it can be suggested that NO donors, including the naturally occurring NO donors’ polyamines, can trigger autophagy by inducing nitrosative and oxidative stress in a similar way to general prooxidants [49]. Polyamines play a dual role in the regulation of redox homeostasis, as they are both a source and a “trap” of ROS, which, in turn, can affect the process of autophagy [12]. Another example of the fine control by NO of some ATG proteins and other proteins involved in autophagic cascades could be NO-mediated posttranslational modifications, such as S-nitrosylation. One more mechanism is likely to be related to cellular energy metabolism. Some aggressive NO donors, such as nitrite, SNP, and spermine at toxic concentrations, can inhibit mitochondrial activity and decrease mitochondrial potential, which are prerequisites for the induction of autophagy in animal cells. In contrast, mild NO donors, such as nitrate and spermine at physiological concentrations, can trigger autophagy-activating pathways involving GADPH and SnRK. As a result, mild NO donors may improve plant performance by triggering autophagy and removing oxidized and damaged cellular constituents without disrupting energy metabolism. Taken together, the results suggest that by triggering autophagy in stressed plant cells, NO can facilitate survival and improve plant performance.

## Figures and Tables

**Table 1:** Effects of NO donors and spermine on the redox status of the roots, the index of membrane stability (IMS), and the rates of respiration.

Treatment	Concentration	H_2_O_2_, μmol g^−1^ FW	Lipid Peroxidation, μmol g^−1^ FW	IMS, a.u.	Respiration Rates, μg h^−1^ g^−1^ FW
Control	-	2.6 ± 0.3	6.2 ± 0.3	72.9 ± 2.5	450 ± 44
KNO_3_	10 mM	3.1 ± 0.6	6.6 ± 0.4	63.5 ± 6.6 *	480 ± 81
KNO_2_	1 mM	5.7 ± 0.8 *	11.8 ± 0.4 *	64.1 ± 5.5 *	261 ± 86
SNP	10 μM	4.8 ± 0.4 *	8.2 ± 0.5 *	63.2 ± 2.6 *	290 ± 81
Spermine	1 μM	4.7 ± 0.7 *	6.1 ± 0.1	73.2 ± 2.4	515 ± 40
Spermine	10 μM	4.3 ± 0.4 *	6.2 ± 0.7	62.7 ± 3.3 *	434 ± 33
Spermine	100 μM	8.6 ± 0.9 *	10.8 ± 0.7 *	61.5 ± 4.1 *	328 ± 51

* *p* ≤ 0.05.

## Data Availability

All relevant data can be found within the manuscript and its Supplementary Materials.

## Supplementary Materials

The following supporting information can be downloaded at https://www.mdpi.com/article/10.3390/antiox12091655/s1, Figure S1: LysoTracker Red dotted fluorescence corresponding to the relative number of autophagosomes per mm^2^ in wheat roots treated with NO donors, spermine and cPTIO. Figure S2: Ultrastructure of autophagosomes (top) and mitochondria (bottom) in spermine treated roots (3 h). Figure S3: Effects of NO donors and spermine on autophagosome formation and cell viability. Top two rows: Visualization of autophagosomes by LysoTracker Red. Figure S4: Western-blot quantification of ATG4 and ATG8 protein accumulation in the wheat roots treated with NO donors and spermine. Figure S5: Relative TMRM dependent fluorescence in wheat roots treated with NO donors and spermine. Figure S6: Digital quantification of the relative activity of mitochondrial complexes IV, III_2_ and supercomplex III_2_+IV. Table S1: List of primers used for qPCR analysis and construction of vector mRFP-TaATG8c. Table S2: Statistical analysis (two-way ANOVA, OriginPro9) of the effects on gene expression of time of exposure and concentrations of spermine.
