# Supplementary material for: Nitric Oxide Induces Autophagy in Triticum aestivum Roots
Source: Antioxidants (Basel). 2023 Aug 22;12(9):1655. doi: 10.3390/antiox12091655 (PMC10525912; doi:10.3390/antiox12091655)
Supplement: Supplementary file 1 [file antioxidants-12-01655-s001.zip › antioxidants-2534527-supplementary.pdf]

Supplementary Material to the manuscript by Minibayeva et al. “Nitric oxide induces autophagy in *Triticum aestivum* roots”

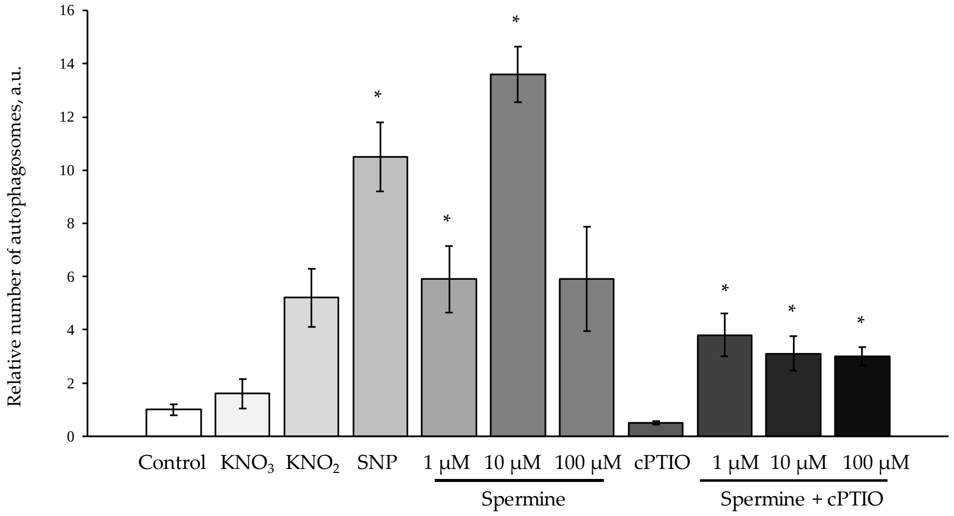

Figure S1. LysoTracker Red dotted fluorescence corresponding to the relative number of autophagosomes per mm^2 in wheat roots treated with NO donors, spermine and cPTIO. The number of autophagosomes in the control was considered as equal to 1. The data are expressed as the mean ± SD ($n = 3$). Asterisk denotes significant difference between control and treatments according to Student *t*-test ($P < 0.05$).

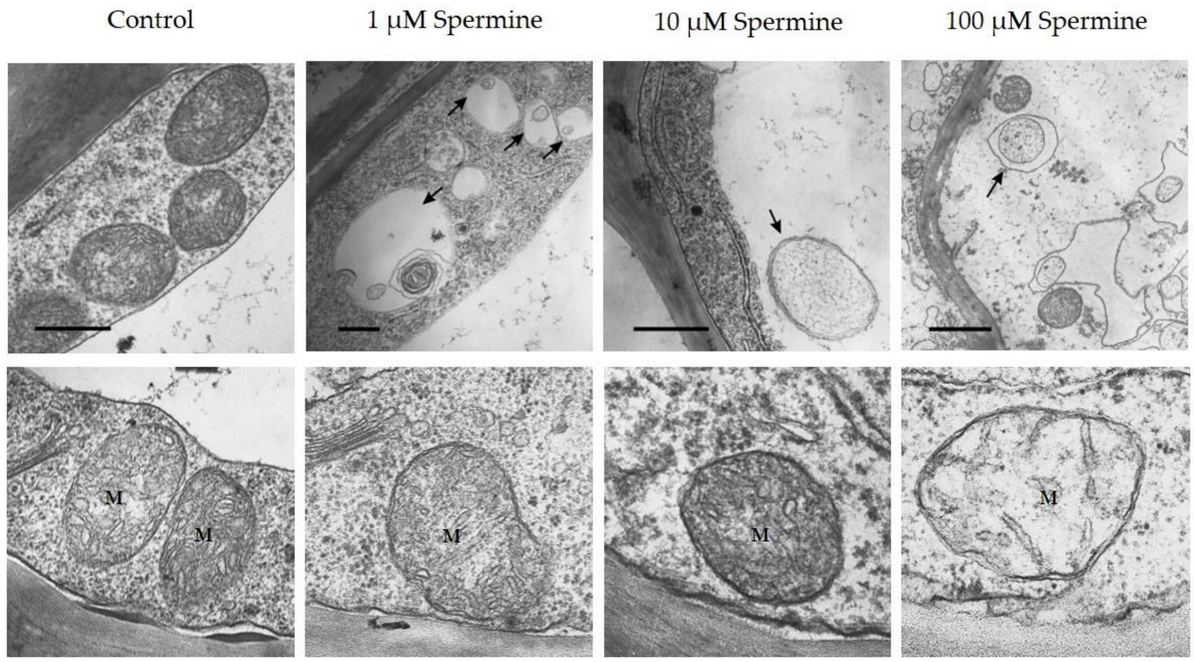

Figure S2. Ultrastructure of autophagosomes (top) and mitochondria (bottom) in spermine treated roots (3 h). Control and 1 μM spermine (bar = 50 μm), 10 and 100 μM spermine (bar = 100 μm). The arrows indicate autophagosomes. M – mitochondrion.

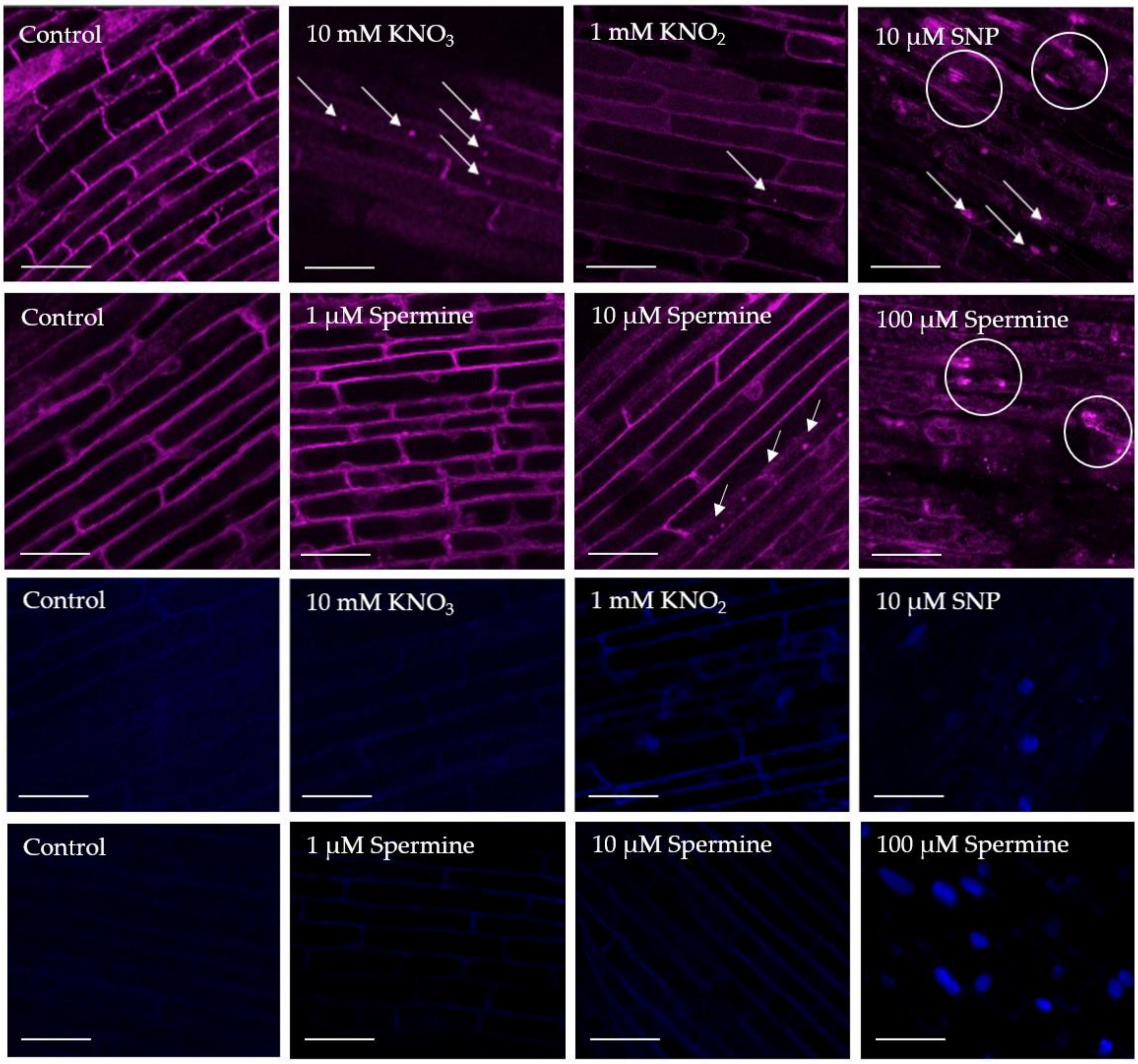

Figure S3. Effects of NO donors and spermine on autophagosome formation and cell viability. Top two rows: Visualization of autophagosomes by LysoTracker Red. The arrows indicate autophagosomes, the circles indicate LysoTracker Red positive conglomerates. Bottom two rows: Visualization of dead cells by propidium iodide (PI, λ_{ab} 485 nm / λ_{em} 610 nm, bar = 50 μm).

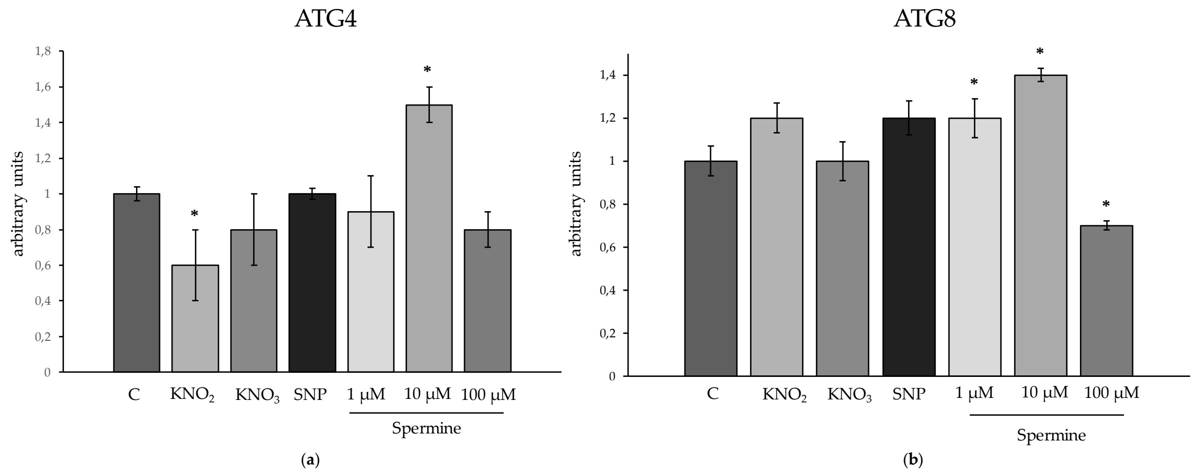

Figure S4. Western-blot quantification of ATG4 and ATG8 protein accumulation in the wheat roots treated with NO donors and spermine. Data represent the mean ± SD (n=3). Asterisk denotes differences significant at $P \leq 0.01$ according to the Student's *t*-test between control and treatments.

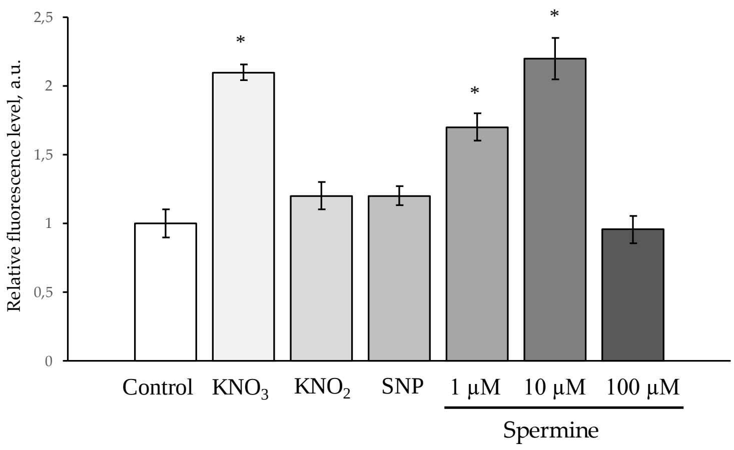

Figure S5. Relative TMRM dependent fluorescence in wheat roots treated with NO donors and spermine. The data are expressed as the mean ± SD (n = 3), asterisk denotes significant difference between control and treatments according to Student *t*-test ($P < 0.05$).

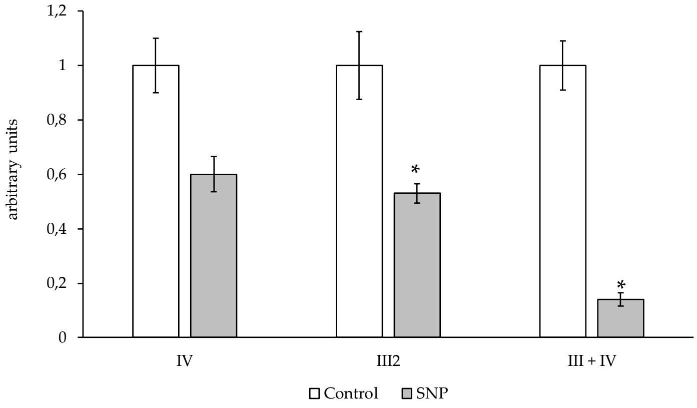

Figure S6. Digital quantification of the relative activity of mitochondrial complexes IV, III_2 and supercomplex III_2+IV. Data represent the mean ± SD (n=3). Asterisk denotes significant difference between control and sodium nitroprusside (SNP) according to Student *t*-test ($P < 0.05$).

Table S1. List of primers used for qPCR analysis and construction of vector mRFP-TaATG8c

**Primer name**	**ID gene**	**Sequence 5'–3'**
ATG1	Traes_4BL_A7AE389EE	F: CCTGAGAGTAATGCCCCAAT<br>R: CGTCCTTCACCAATCTTGC
ATG4	FJ750846.1	F: CTAGTGATGTCAACTGGGGCTGC<br>R: GATCCTTGTATGTTCTGGGTCAGATG
ATG8 af	TRIAE_CS42_2AL_TGACv1_093258_AA0276020.1	F: CGCATAAGGGAGAAGTACTCTGACA<br>R: CAAAGATGAAGATCGCCTTCTCA'
ATG8 g	TRIAE_CS42_2AS_TGACv1_114996_AA0370340.2	F: GGCTGATAAGTCTGATGTCCCG<br>R: GAAGCAGTCGGTGGCAAGG
ATG8 h	TRIAE_CS42_5AL_TGACv1_374995_AA1213590.1	F: GGTTCCGTGTGACATGCCA<br>R: GCCATCCTGCTTATCTTTGTACG
SNRK beta/ gamma subunit	TRIAE_CS42_2AL_TGACv1_094408_AA0297150.1	F: CCAATCATCTATTCATCGTCA<br>R: CGCAACATAGCCAATGGAT
SNRK beta 3 subunit	TRIAE_CS42_1DS_TGACv1_081316_AA0259820.1	F: GGCAGGGATGACCATGAA<br>R: CGGGAAGCTCAGCAGTGT
SNRK beta 1/2 subunit 1L	TRIAE_CS42_1AL_TGACv1_001781_AA0035120.2	F: CGGATGAATCCACCAACAAT<br>R: GACCTCTCTAACACCTTCCTTGA
SNRK beta 1/2 subunit 4L	TRIAE_CS42_@DS_TGACv1_178522_AA0597000.1	F: GTTCCTGAAAGTGTTGAAAGTGT<br>R: GGTGGTTGAGGACGATGTG
SNRK alpha subunit 1L	TRIAE_CS42_1AL_TGACv1_001183_AA0026620	F: CAGTATGGTTCTCCAGGGTTT<br>R: GCCATCAGCTTCAATTATAGCA
SNRK alpha subunit 3L	TRIAE_CS42_3AL_TGACv1_195454_AA0649780.1	F: GGAAATCGGCAACAACCATA<br>R: CCAGGTCGTCGGTTTCAATA
ATG8c XmaI	-	F: GGACCCGGGATGGCGAAGAGCTCGTTCAAG
ATG8c XbaI	-	R:CAATCTAGATTAGTGGTGGTGATGGTGATGATGGTG
mRFP BamHI	-	F: CTGGATCCACCATGGCCTCCTCCGAGGAC
mRFP XmaI	-	R: CACCCCGGGTCCAGCTCCAGAAGGAGGT

Table S2. Statistical analysis (two-way ANOVA, OriginPro9) of the effects on gene expression of time of exposure and concentrations of spermine. Differences were significant at (*) $P \leq 0.05$, (**) $P \leq 0.01$.

**Genes**	**Time**	**Concentration**	**Interaction**
TaATG1 (1)	*	*	*
TaATG3a	**	*	*
TaATG4	ns	*	ns
TaATG5	ns	*	ns
TaATG5a	**	*	*
TaATG6b	**	**	**
TaATG6c	**	**	**
TaATG7	*	ns	ns
TaATG8 af	ns	ns	ns
TaATG8 g	ns	**	ns
TaATG8 h	ns	ns	ns
TaATG10	*	*	*
TaATG12 b	**	*	*
TaATG13 a	**	*	*
TaAtg13g	**	*	*
TaGAPC	ns	ns	ns
TaGAPDH (1)	*	ns	ns
TaGAPDH (4)	ns	ns	ns
TaGAPDH (6)	*	ns	ns
TaGAPDH (9)	*	*	*
TaGAPDH (12)	*	ns	ns
TaSnRK1 β/ γ	**	ns	ns
TaSnRK1 β3	**	ns	ns
TaSnRK1 β1/2 (1L)	*	*	*
TaSnRK1 β1/2 (4L)	*	ns	ns
TaSnRK1 α (1L)	*	ns	ns
TaSnRK1 α (3L)	*	*	*

ns – not significant
